# ICU Length of Stay and Factors Associated with Longer Stay of Major Trauma Patients with Multiple Rib Fractures: A Retrospective Observational Study

**DOI:** 10.1155/2022/6547849

**Published:** 2022-03-01

**Authors:** Hesham S. Abdelwahed, F. Eduardo Martinez

**Affiliations:** ^1^Intensive Care Unit, John Hunter Hospital, Newcastle, New South Wales, Australia; ^2^School of Medicine and Public Health, University of Newcastle, Newcastle, Australia

## Abstract

**Background:**

Chest injury with multiple rib fractures is the most common injury among major trauma patients in New South Wales (23%) and is associated with a high rate of mortality and morbidity. The aim of this study was to determine the intensive care unit (ICU) length of stay (LOS) among major trauma patients with multiple rib fractures and to identify factors associated with a prolonged ICU LOS.

**Materials and Methods:**

Single-centre, retrospective observational cohort study of adult patients with 3 or more traumatic rib fractures, who were admitted to ICU between June 2014 and June 2019. A comparison was made between patients who stayed in ICU for less than 7 days and those that stay for 7 or more days.

**Results:**

Among 215 patients who were enrolled, 150 (69.7%) were male, the median Injury Severity Score (ISS) was 24 (interquartile range (IQR): 17–32). The median ICU LOS was 4 (IQR: 2–7) days and the average ICU LOS was 6.5 (SD 8.5; 95% CI 5.3–7.6) days. The median number of rib fractures was 6 (IQR: 5–9) and 76 (35.3%) patients had a flail chest. Patients who stayed longer than 7 days in ICU had higher ISS, higher APACHE-II score, greater number of rib fractures, higher rate of lung contusions, and required more respiratory support of any type.

**Conclusions:**

ISS, number of rib fractures, lung contusion, and flail chest were associated with prolonged ICU LOS in patients with traumatic multiple rib fractures.

## 1. Introduction

Major trauma can be defined as having an Injury Severity Score (ISS) of more than 12 [1]. In the state of New South Wales, Australia, there are about 4,000 severe polytrauma hospital admissions per year [2]. About one in two of these patients have chest injuries, and as many as one in four of these patients have chest injuries with three or more rib fractures [2]. Multiple rib fractures are associated with high morbidity and mortality [3]. Morbidity presents mostly as thoracic complications that can include atelectasis, hospital-acquired pneumonia (HAP), and respiratory failure [4]. With higher injury severity, morbidity occurs more often. Flail chest, which is the paradoxical motion on clinical examination that occurs when there are 3 or more continuous ribs fractured in 2 or more places [5], is associated with worse outcomes and a higher incidence of pulmonary morbidity [6, 7].

Multiple rib fractures increase ICU length of stay (LOS) as well as hospital LOS [8]. It is important for healthcare systems to look at lengths of stay because they have a significant impact on healthcare costs [9].

Overall, the average ICU LOS of major trauma patients with multiple rib fractures varies widely and has been described as between 3.5 and 7.5 days. This seems to depend on the severity of injuries, the presence of concomitant injuries, and whether chest injuries with multiple rib fractures have received surgical fixation or not [4, 10–12]. Previous international reports have identified factors like the number of ribs fractured, the presence of flail chest, and an age of more than 65 years as associated with prolonged ICU LOS for severely injured major trauma patients [10, 13, 14]. Factors such as undergoing surgical fixation of rib fractures with a flail chest, the use of multimodal analgesia that includes regional techniques with local anaesthetics, and the implementation of multidisciplinary clinical management pathways have been associated with a shorter ICU LOS [15–21].

Identifying factors associated with a prolonged ICU LOS could help clinicians anticipate which patients are likely to require a longer stay in ICU and thereby allocate resources more efficiently. It could also help medical teams target and treat more aggressively the modifiable factors that are associated with longer ICU stays.

Determining ICU LOS for different patient groups, including trauma patients, is helpful because it can be used for benchmarking outcomes through a measure that is readily available and provides many insights [22].

The main aim of this study was to determine the median ICU LOS of major trauma patients with multiple rib fractures admitted to an Australian regional, tertiary-referral, trauma ICU. The secondary aim was to determine the rate of modifiable and nonmodifiable risk factors that occurred in patients who had an ICU LOS of less than 7 days versus patients who had an ICU LOS of 7 or more days.

## 2. Methods

This is a single-centre, retrospective, cohort observational study. It has been undertaken at John Hunter Hospital (JHH), which is a tertiary-referral, major trauma centre with an average of about 600 major trauma admissions per year. JHH services a large rural population so close to 30% of those major trauma admissions arrive by medical retrieval services [2].

All patients with multiple rib fractures, with or without flail chest, who were admitted to the ICU at JHH between June 2014 and June 2019, were screened. The International Statistical Classification of Diseases and Related Health Problems 10th Revision (ICD-10) Version for 2010 [23], codes S22.3, S22.4, and S22.5, were used to select patients. Archived digital medical records were reviewed by the investigators. Approval from the local human research ethics committee was obtained before data collection began (AU201909-17).

The inclusion criteria were as follows: (1) adult patients 18 years or older; (2) major trauma defined as ISS more than 12; (3) three or more rib fractures; and (4) non-penetrating chest trauma. The exclusion criteria were as follows: (1) patients younger than 18 years of age; (2) nontraumatic rib fractures; (3) patients with traumatic brain injury (TBI) on admission, defined as an alteration of brain function or evidence of brain pathology caused by external force [24]; and (4) pregnant patients.

The primary outcome was to investigate ICU LOS among major trauma patients with three or more rib fractures who were admitted to the ICU of a major trauma centre in Australia. The secondary outcomes were as follows: (1) to divide and quantify patients into those who had an ICU LOS of less than seven full days and those who had an ICU LOS of seven or more full days and (2) to compare the groups to identify factors associated with a longer ICU LOS. The decision to divide the groups based on whether patients stayed in ICU for more or less than seven full days was made due to the ease of comparing full weeks rather than fractions. This number still lies between what is reported in the literature as average ICU LOS for this cohort of patients [25].

Data were collected from the hospital's digital medical records system. Data was collected on demographics; mechanism of trauma; Injury Severity Score (ISS) at hospital admission; acute physiology and chronic health evaluation II (APACHE-II) score at ICU admission; number of rib fractures; associated injuries; respiratory support use, including high-flow nasal prongs (HFNP), noninvasive ventilation (NIV) and invasive mechanical ventilation (IMV); regional pain management interventions, including type and location; the incidence of HAP [26], defined as pneumonia that occurs 48 hours or more after hospital admission, and ventilator-acquired pneumonia (VAP) [26], defined as pneumonia that occurs 48 hours or more after endotracheal intubation; the rate of flail segments, defined as the confirmed radiological finding on the CT scan report of three or more contiguous ribs fractured in two or more places, which is used as a surrogate for clinical flail chest due to the retrospective nature of this study; and management of rib fractures, either with surgical fixation or conservative management.

Comparisons were made to determine whether there were differences in the rate of nonmodifiable risk factors, including (1) age, (2) gender, (3) APACHE-II score, (4) ISS, (5) number of rib fractures, (6) rate of flail segments identified by CT scan, (7) rate of flail segments, (8) rate of HAP or VAP, and (9) rate of ICU mortality. Comparisons were also made between the rate of modifiable risk factors, including (1) rate of use of regional blocks, (2) rate of use of HFNP, (3) rate of use of NIV, (4) rate of use of IMV, (5) rate of surgical rib stabilization, and (6) rate of use of tracheostomy.

Descriptive statistics are presented as numerator and denominator, with percentage in parenthesis. The data collected were considered nonparametric, and outcome measures were expressed as medians with interquartile ranges. Statistical analysis was performed using the Prism 9 software. Mann–Whitney *U* test or chi-square test were used for comparison between groups depending on the type of variables. Statistically significant differences are considered when *P* < 0.05.

## 3. Results

**Figure 1:**
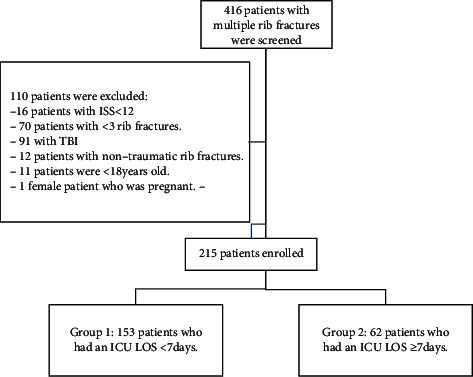
Screening and analysis flowchart.

During the study period June 2014 to June 2019, there were 416 patients admitted to ICU with multiple rib fractures who were screened for inclusion. Of these, 215 patients met all of the inclusion criteria and had none of the exclusion criteria and were included in the analysis (Figure 1).

There was an average of 43 major trauma patients with chest injuries that had multiple rib fractures as the primary reason for admission to ICU per year. The most common causes of chest trauma with multiple rib fractures were traffic accidents with 140/215 (61.1%), which include “car versus car,” “car versus stationary object,” “pedestrian versus car,” or “motorbike accident.” These were followed by falls with 51/215 (16.7%) of patients. Of the patients in the cohort 137/215 (63.7%) had a pneumothorax, 118/215 (54.9%) had sustained lung contusions, and 108/215 (50.2%) had a haemothorax.

Demographic and other characteristics of the cohort are presented in Table 1.

In this cohort, the median ICU LOS was 4 (IQR 2–7) days, and the median hospital LOS was 15 (IQR 9–27) days. In this study, 153/215 (71.2%) of patients had an ICU LOS of less than seven days and 62/215 (28.8%) had an ICU LOS of seven or more days. Comparison between patients with ICU LOS of less than seven days and those with ICU LOS of seven or more days is presented in Table 2.

## 4. Discussion

ICU LOS can be used as a tool for benchmarking a unit's performance because it is easy to measure, is clinically relevant, and can be used to provide insight about the efficiency of a unit [22]. The measures of central tendency for ICU LOS of this cohort (median 4 (IQR 2–7) and mean 6.5 (SD 8.5; 95% CI 5.3–7.6) days) where quite similar to what has been described in national [2, 27] and international reports [10, 25] for major trauma patients with similar median ISS.

The secondary outcome of this study was to compare patients who had a shorter versus a longer ICU LOS. This was done to determine whether there are modifiable factors that can be targeted. There were differences between the two groups in terms of injury severity, concomitant injuries, treatment, and outcomes.

When comparing age and gender, there was no difference between groups, but the APACHE-II scores on ICU admission, ISS scores on hospital admission, and the number of broken ribs were all statistically higher in the group with the longer ICU LOS. APACHE-II scores were used because they are readily available from the electronical medical files at our hospital, and they are widely used throughout the world, even though APACHE-III or Sequential Organ Failure Assessment (SOFA) scores could also have been used [28]. It is worth noting that the median ISS of the cohort was higher than other reports of trauma patients in ICU [29–31]. It is difficult to determine exactly why that is when only looking at this dataset. The reason is likely multifactorial and in part due to JHH having a highly functioning trauma ward which is able to manage severely injured patients who do not require a high level of organ support. These findings argue that markers of injury severity are reliable when used by clinicians to predict a longer ICU LOS and allocate resources appropriately. ISS is known to have a linear relationship with ICU LOS. Previous reports have described how the average ICU LOS increased from 3.3 days to 11.4 days when the ISS went from being between 13 and 15 to between 41 and 75 [2, 32]. The number of rib fractures has also been described as associated with prolonged ICU and hospital LOS and that was found here as well [4]. Moreover, sustaining 7 or more rib fractures is known to be independently associated with an increased incidence of pneumonia and increased ICU LOS [10, 13, 33]. Holcomb et al. found that 4 or more fractured ribs were associated with worse outcomes and prolonged ICU and hospital LOS, even in younger populations [34].

There has been some debate as to whether lung contusions increase ICU LOS. Multiple studies [7, 35, 36] have reported that lung contusions were associated with worse outcomes and prolonged ICU and hospital LOS. Fokin et al. and Dhar et al. did not find differences in ICU LOS with or without lung contusions in major trauma patients [37, 38]. The findings of this study can add weight to their argument that lung contusions do not increase ICU LOS.

In this study, patients who had both chest injuries and TBI were excluded from the analysis. Concurrent TBI in major trauma patients with multiple rib fractures has been reported to occur in between 15 and 26% of cases, and the combination of both has been shown to lead to worse outcomes [4, 7, 39]. Excluding TBI patients will hopefully provide a clearer view of factors that contribute to longer ICU LOS that is specifically related to the chest injury.

In our centre, there is no fixed clinical management pathway for the treatment of multiple rib fractures in trauma patients. Instead, multidisciplinary teams made up of emergency physicians, trauma teams, anaesthetists, and ICU teams review and discuss patients on a case-by-case basis and achieve consensus about what would be the best way to treat each individual patient as they arrive in the emergency department. The use of regional blocks including what type of block and the timing of the block are among the issues discussed, along with the potential risks and benefits of surgical rib fixation, and the disposition of the patient after leaving the emergency department.

The use of regional analgesia was the same in both groups. Regional blocks are widely utilised on major trauma patients with multiple rib fractures because they are readily available and minimally invasive.

The decision to admit a patient to the ICU is made by the intensivist. It is based on whether there is a need for organ support, close monitoring and observation, or presence of risk factors for deterioration.

Patients in the longer ICU LOS group required a significantly higher rate of all types of respiratory support. This difference was more pronounced with IMV, where almost 90% of patients who required IMV had a longer ICU LOS. If it is at all possible, and often it is not possible for different reasons, avoiding IMV through noninvasive respiratory support and aggressive analgesia strategies might help patients require a shorter ICU stay.

A statistically significant higher percentage of patients in the group with longer ICU LOS had flail segments as well as underwent rib fixation. At JHH, the decision to offer surgical stabilization is at the discretion of the trauma surgeon. It is made after considering patient factors and injury factors. Whether surgical rib stabilization shortens ICU LOS is still being debated. It is likely associated with the presence or absence of a flail chest. Fokin et al. have reported longer ICU LOS for those who had surgical rib stabilization [37]. In 3 subsequent meta-analyses, surgical rib stabilization of multiple rib fractures with flail chest was associated with a decrease in the ICU LOS [15, 16, 40]. Additionally, Xiao et al. have reported that surgical rib fixation shortened ICU LOS in patients with multiple rib fractures and flail chest, but it did not in those without flail chest [41].

When looking at outcomes, we can see there was a significantly higher rate of nosocomial pneumonia cases in the group that stayed longer in ICU. It is feasible that higher injury severity could lead to more time on a ventilator, which could lead to a higher incidence of VAP, which could then lead to a longer ICU LOS [42]. This is one of the reasons why critical care clinicians endeavour to liberate patients from ventilators as soon as possible and through so many ways, such as with aggressive physiotherapy involvement, the use of judicious sedation practices, and regional analgesic techniques [21]. The total number of tracheostomies performed was low. In our centre, tracheostomies are mostly used after 10 days of IMV, and the ICU LOS and the time of IMV here were both much less than that [43].

There were differences between the 2 groups in terms of injury severity, concomitant injuries, and treatment received, but mortality was similar. Multidisciplinary approaches have been proven to be effective in many settings and likely have a large role to play here as well [18, 44].

### 4.1. Strength and Limitations

This study has important strengths. It includes major trauma patients with multiple rib fractures that had a high level of acuity. It provides a detailed look at the characteristics and outcomes at this level of severity. This data yielded similar results to previous reports, which would indicate that it is accurate. The limitations are that it is a single-centre, retrospective, and observational study, so it is possible that there has been selection bias and missing data, among other problems associated with retrospective studies. Another potential limitation is that the type of trauma patients who present to JHH might be mostly those involved in high-speed motor vehicle accidents that occur on long stretches of highway. The insights that this study is looking to provide might not be applicable to a more metropolitan setting, but we feel is still worth sharing.

## 5. Conclusion

In this study of major trauma patients with multiple rib fractures with high severity scores, and admitted to a tertiary-referral ICU, the ICU LOS was similar to national and international centres. Nonmodifiable factors such as a high ISS and APACHE-II scores, a higher number of rib fractures, and the presence of lung contusions and flail chest segments were associated with an ICU LOS of 7 or more days. Modifiable factors such as the need for any type of respiratory support, surgical rib stabilization, and tracheostomies were also associated with a longer ICU LOS. More studies are needed to help delineate strategies that can decrease the ICU LOS of patients who are more severely injured.

## Figures and Tables

**Table 1:** Demographic characteristics and interventions of the cohort.

Basic characteristic	*N* = 215
Age, median (IQR)	59 (45–70)
Male gender	150/215 (69.7%)
APACHE-II score, median (IQR)	11 (8–16)
ISS score, median (IQR)	24 (17–32)
GCS, median (IQR)	15 (4–15)
Mechanism of trauma	
(1) Traffic accident, percentage	140/215 (65.1%)
(i) Car	104/215 (48.3%)
(ii) Motorbike	36/215 (16.7%)
(2) Fall	51/215 (23.7%)
(3) Others	24/215 (11.1%)
No. of rib fractures, median (IQR)	6 (5–9)
Flail segments	76/215 (35.3%)
Lung contusion	118/215 (54.9%)
HAP/VAP	26/215 (12.1%)
HFNP	112/215 (52.1%)
NIV	43/215 (20%)
IMV	97/215 (45.1%)
Duration of IMV, days (median (IQR))	0 (0–4)
Regional analgesia	111/215 (51.6%)
Surgical rib stabilization	28/215 (13%)
Tracheostomy	7/215 (3.3%)
ICU LOS, days (median (IQR))	4 (2–7)
Hospital LOS, days (median (IQR))	15 (9–27)
Mortality rate	15/215 (7%)
ICU readmission rate	11/215 (5.1%)

IQR: interquartile range; APACHE-II: acute physiology and chronic health evaluation II; ISS: injury severity score; No: number; GCS: Glasgow coma score; IMV: invasive mechanical ventilation; NIV: noninvasive ventilation; HFNP: high-flow nasal prongs; HAP: hospital-acquired pneumonia; VAP: ventilator-associated pneumonia; TBI: traumatic brain injury; ICU: intensive care unit; LOS: length of stay.

**Table 2:** Comparison between patients with ICU LOS of less than 7 days and patients with ICU LOS of 7 or more days.

	ICU LOS <7 days (*N* = 153)	ICU LOS ≥7 days (*N* = 62)	*P* value and OR (95% CI)
ICU LOS, median (IQR)	3 (2–4)	11 (8–17.3)	*P* < 0.0001

*Nonmodifiable*			
Age, median (IQR)	57 (43–70)	62 (48.3–70.3)	*P* = 0.16
Male gender	106/153 (69.3%)	44/62 (71%)	*P* = 0.87; OR: 0.92 (95% CI: 0.48 to 1.72)
APACHE-II, median (IQR)	9 (7–14)	16 (11–21)	*P* < 0.0001
ISS, median (IQR)	24 (17–29)	29 (20–36)	*P* < 0.002
No. of rib fractures, median (IQR)	6 (4–9)	7 (5–10)	*P* = 0.02
Flail segments	43/153 (30.1%)	30/62 (48.4%)	*P* = 0.01; OR = 0.46 (95% CI: 0.26 to 0.86)
Lung contusions	75/153 (49%)	30/62 (48.4%)	*P* > 0.99; OR: 1.03 (95% CI: 0.57 to 0.1.87)
HAP/VAP	3/153 (0.02%)	23/62 (37.1%)	*P* ≤ 0.0001; OR: 0.05 (95% CI: 0.02 to 0.17)
ICU mortality	9/153 (0.06%)	6/62 (0.1%)	*P* = 0.38; OR: 0.58 (95% CI: 0.21 to 1.74)

*Modifiable*			
Regional block	74/153 (48.4%)	37/62 (60%)	*P* = 0.18; OR = 0.63 (95% CI: 0.35 to 1.15)
HFNP	69/153 (45.1%)	43/62 (69.4%)	*P* = 0.005; OR: 0.42 (95% CI: 0.23 to 0.76)
NIV	25/153 (16.3%)	18/62 (29%)	*P* = 0.04; OR: 0.48 (95% CI: 0.24 to 0.96)
IMV	42/153 (12.1%)	55/62 (88.7%)	*P* ≤ 0.0001; OR: 0.05 (95% CI: 0.02 to 0.11)
Surgical stabilization	12/153 (7.8%)	16/62 (25.8%)	*P* = 0.001; OR: 0.30 (95% CI: 0.14 to 0.60)
Tracheostomy	0/153 (0.5%)	7/62 (11.3%)	*P* ≤ 0.0001; OR: 0.00 (95% CI: 0.00 to 0.21)

*N*: number; ICU: intensive care unit; LOS: length of stay; ISS: injury severity score; IQR: interquartile range; APACHE-II: acute physiology and chronic health evaluation II; IMV: invasive mechanical ventilation; NIV: noninvasive ventilation; HFNP: high-flow nasal prongs; CI: confidence interval; TBI: traumatic brain injury; HAP: hospital-acquired pneumonia; VAP: ventilator-associated pneumonia.

## Data Availability

The data used to support the findings of this study are included within the article.
